# Trust in regional politicians and mortality: A population-based prospective cohort study

**DOI:** 10.1016/j.pmedr.2023.102189

**Published:** 2023-04-01

**Authors:** Martin Lindström, Mirnabi Pirouzifard

**Affiliations:** Social Medicine and Health Policy, Department of Clinical Sciences and Centre for Primary Health Care Research, Lund University, S-205 02 Malmö, Sweden

**Keywords:** Institutional trust, Political trust, Generalized trust in other people, Social capital, Mortality, Cardiovascular mortality, Cancer mortality, Sweden

## Abstract

The aim was to study associations between trust in regional politicians responsible for the healthcare system and mortality in survival analyses. A public health survey in southern Sweden with a 54.1% response rate based on a postal questionnaire and three postal reminders was conducted in 2008. The baseline survey was linked to 8.3-year follow-up all-cause, cardiovascular (CVD), cancer and other causes mortality register data. The present prospective cohort study includes 24,699 respondents. Relevant covariates/confounders from the baseline questionnaire were included in the multi-adjusted models. Hazard rate ratios (HRRs) of all-cause mortality were consistently lower for the rather high trust and not particularly high trust respondent categories compared to the very high trust reference category. CVD, cancer and other causes mortality did not display statistically significant results, but all contributed to the significant patterns for all-cause mortality. In some political and administrative settings with longer queueing times for investigation and treatment of some medical conditions including some cancer and CVD diagnoses than officially affirmed, rather high and not particularly high trust in politicians responsible for the healthcare system may be associated with lower mortality compared to the very high trust group.

## 1 Introduction

Political trust is closely connected with democratic governance. Political trust builds on information, reason and debate. It is only possible to fully measure political trust in democracies. Already in 1690, political philosopher John Locke (1632–1704) reflected on trust between citizens as well as trust between citizens and the government and public authorities. His reflections resulted in a contract theory defining government power and authority as a result of an original contract signed by the people, with the ultimate meaning that all power originates from the people (Locke, 1988/1690). For Locke, the idea of an original contract was synonymous with the notion of political trust (Dickinson, 1977). Political trust and political support exist at different levels of political community, regime and authority (Easton, 1965a, Easton, 1965b, Easton, 1975, Norris, 1999). The fact that most constituents/voters often lack complete information regarding political issues and decisions adds to the complex nature of political trust. According to Rudolph (2017), trust in politicians at any level of the political system, whether national, regional or municipal, may be regarded as a behavioral heuristic and as a cognitive shortcut for citizens to make judgements on complex issues in the absence of complete information. Previous literature also suggests that trust can be blind and unconditioned (Myskja et al., 2020), which questions the purely positive meaning of trust. While high or medium levels of trust may be better than no trust at all and even stimulate questioning and debate, very high and unconditioned trust may have negative effects (Uslaner, 2002). This study aims to investigate associations between political trust and mortality. It also aims to investigate whether very high levels of political trust may increase mortality relative to moderate trust, given the suggestion in the literature that unconditioned trust can have adverse effects.

In Sweden, political trust in regional politicians responsible for the healthcare system is primarily connected with the current status and long-term development of the healthcare system. Sweden is divided into 21 independent regions with the right to tax the population in order to finance, organize and provide healthcare. These regions (previously county councils) constitute the backbone of the Swedish healthcare system since 1862. *Region Skåne* is such a region. The regions are governed according to Swedish law sanctioned by the national parliament (*Riksdag*) but with their own elections every fourth year on the same day as the national and municipal elections.

Political trust in general and more specifically political trust in politicians responsible for the healthcare system constitutes a form of asymmetrical trust. Trust in healthcare providers entails an asymmetrical relationship between the caregiver and the patient in the form of an information and power gradient (Freidson, 2001). Such asymmetrical trust may be both personal between individual caregivers and patients, and institutional in relation to the healthcare system and responsible political leadership (LoCurto and Berg, 2016). Lack of trust in healthcare providers may originate in the very top of the administrative and political leadership of the healthcare system due to lack of adequate resources or suboptimal distribution of resources (Topp and Chipukuma, 2016). Asymmetrical trust is part of the social capital concept (Coleman, 1990, Putnam, 1993). Social capital is defined as social structures, social participation, civic participation, norms of mutual aid and reciprocity, generalized (horizontal) trust in other people without a power gradient and institutional, vertical or asymmetrical trust across a power gradient, which all are assumed to increase cooperation and collective action (Putnam, 1993). Institutional/asymmetrical trust specifically entails citizens’ trust in political and public institutions (Narayan, 2002, Narayan and Cassidy, 2001).

Just as social participation and generalized trust in other people without a power gradient are significantly associated with better health (Lindström, 2004) and lower mortality (Kawachi et al., 1999, Lindström et al., 2021), asymmetrical institutional (vertical) trust may be associated with health. A Swedish study of institutional trust as an index measure integrating trust in healthcare, school system, social welfare services, labor office, social insurance office, police, court of law, parliament, politicians at regional and municipal level found that low institutional trust was associated with harmful alcohol consumption (Ahnquist et al., 2006). A study from southern Sweden found that low institutional trust was associated with smoking and inversely associated with smoking cessation (Lindström and Janzon, 2007). More specifically, self-rated trust in the healthcare system is regarded internationally as a valid measure for the evaluation of healthcare performance (Straten et al., 2002, Chang et al., 2013). Trust in the healthcare system has a wide array of positive effects on health including increased motivation and compliance with prevention, propensity to seek health services when needed, compliance with medication and other treatments, improved placebo effect, increased compliance with medical advice and acceptance of medical suggestions (Lewandowski et al., 2021), and higher self-rated health (Mohseni and Lindström, 2007). In contrast, a previous prospective cohort study has demonstrated adverse results with significantly lower CVD and cancer mortality among respondents with rather high and not particularly high trust in the healthcare system compared to the very high trust reference group. These results were associated with queueing problems in the healthcare system related to cancer and CVD treatment (Lindström and Pirouzifard, 2022).

Studies have shown links between political trust and health compliance (Robinson et al., 2021, Kestilä-Kekkonen et al., 2022) and mortality (Bollyky et al., 2022, Charron et al., 2022) in relation to COVID-19. Cross-sectional studies in Scania have shown that low political trust in national politicians is significantly associated with poor self-reported psychological health (GHQ12) (Lindström and Mohseni, 2009), cannabis smoking (Lindström, 2008a), purchase of illegal liquor (Lindström, 2008b), and poor self-rated health (Mohseni and Lindström, 2008). Still, studies regarding political trust and mortality are rare. The first hypothesis of this study is that lower trust in regional politicians is associated with higher mortality. The second hypothesis is that very high level of political trust may increase mortality relative to the groups with low or moderate trust levels, given the notion of unconditioned trust as a cognitive shortcut (Myskja et al., 2020).

The aim of this study is to investigate associations between trust in regional politicians and all-cause, CVD, cancer and other causes mortality, adjusting for relevant covariates. All independent variables stem from a survey conducted in 2008.

## 2 Material and methods

### 2.1 Study population

The public health survey in Scania 2008 is cross-sectional and based on a stratified sample of the population aged 18–80. The survey was conducted in the autumn of 2008 in Scania (Skåne) in southern Sweden. The initial postal invitation letter included a questionnaire. It was later followed by three postal reminders to non-responders. It was also possible to complete the questionnaire online. A total 28,198 persons participated, which yielded a 54.1% response rate. The survey was performed by *Region Skåne*, which is the regional public authority responsible for the healthcare system in Scania. The 2008 survey questionnaire includes 134 items concerning sociodemographic characteristics, self-rated health, self-rated psychological health, social support, social anchorage, social capital, health-related behaviors, sense of security, health concerns and healthcare utilization items. The random sample was stratified according to age, sex, education and municipality/city part. The stratified sample was generated by *Statistics Sweden* in Stockholm from its national population register. *Statistics Sweden* also created the population weight. The cross-sectional baseline survey data from 2008 was linked to prospective register mortality data accessed from *the National Board of Health and Welfare* (*Socialstyrelsen*).

This study combines the baseline survey data from 2008 to prospective mortality data. Ethical approval was granted from the Ethical Committee (*Etikprövningsnämnden*) in Lund (No. 2010/343).

### 2.2 Dependent variables

Mortality was followed from 27 August-14 November 2008 (exact date depending on registration date of answers from individual respondents) until 31 December 2016 (8.3 years later), or until death. In total, 24,699 participants were included in the present study, 11,245 men and 13,454 women, after exclusion of 3,363 respondents with internally missing values on one or more items from the baseline survey, and 136 participants were lost to follow-up. The Swedish ten-digit person number system facilitates linking the baseline survey data from the 2008 survey with the national causes of death register. A third party (private company) conducted the linkage. The person numbers were deleted prior to delivery to the research group.

All-cause mortality was analyzed, and all-cause mortality was also stratified into the three broad categories cardiovascular (CVD) (I00-I98 according to ICD10), cancer (C00-C97), and other causes (than I00-I98 and C00-C97) mortality. All-cause mortality is the sum of the three broad CVD, cancer and other cause mortality categories.

### 2.3 Independent variables

*Trust in regional politicians* was measured with the question “What trust do you have in regional politicians?” with the alternative answers “Very high trust”, “Rather high trust”, “Not particularly high trust”, “No trust at all” and “Don’t know”. Measurement of political trust often entails 5-alternative (Bangsted et al., 2022, Choi et al., 2022) and 11-alternative (Clench-Aas and Holte, 2021, Schraff, 2021) items. Our political trust item includes the “don’t know” alternative, which is almost required practice in social survey design.

**Fig. 1:**
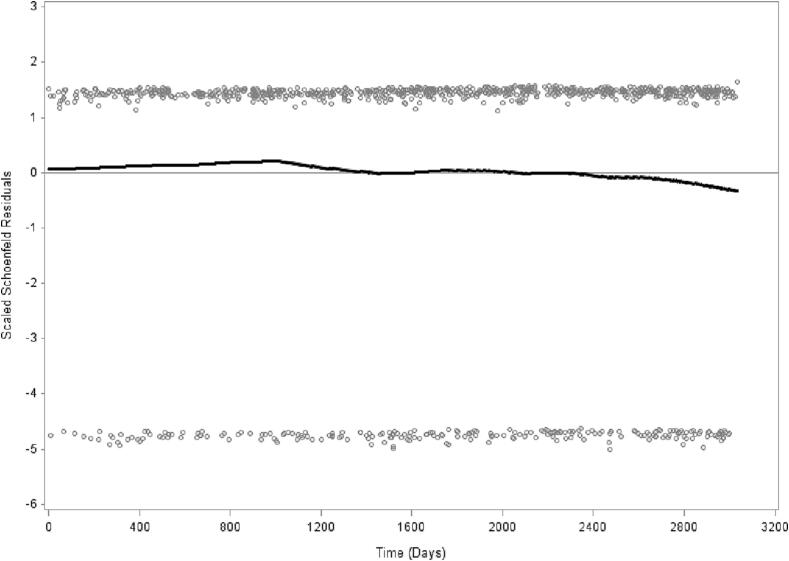
**Schoenfeld residuals for trust in regional politicians and all-cause mortality. Men and women combined. The 2008 public health survey in Scania, Sweden with follow-up 2008**–**2016. Proportionality test:** We performed the proportionality test after removing individuals with the value 5 (5756 individuals) from variable trust in the regional politicians. Also 18,943 persons remained for the proportionality test. Then by using variable trust in the regional politicians we created a new variable with two groups by placing individuals with value 1 and 2 in one group (n = 5261; 27.8%) and those with values 3 and 4 in another groups (n = 13682; 72.2%). In addition, the total number of deaths decreased from 1241 to 986 individuals. The p-value for proportionality based on interaction term between the variable trust in the county council politicians and time of follow-up is 0.412.

Men and women were collapsed in all analyses in Table 1, Table 2, Table 3 and Fig. 1. Analyses in Table 2, Table 3 were adjusted for sex.

**Table 1:** Descriptive characteristics (%) of age (mean age), body mass index (BMI), sex, socioeconomic status (SES),country of birth, chronic disease, low leisure time physical activity (LTPA), smoking, alcohol consumption and generalized trust in other people by trust in regional politicians. The 2008–2016 Public Health Survey of Scania, Sweden. Total population n = 24699 (Men = 11,245 and women = 13,454). **Weighted prevalence.**

		**Trust in regional politicians**					
		**Very high**	**Rather high**	**Not particularly high**	**No trust**	**Don’t know**	p-value
		n = 305	n = 4956	n = 10115	n = 3567	n = 5756	
		1.4%	20.3%	39.3%	14.2%	24.8%	
**Age**, yrs: mean ± SD a		41.8 ± 17.9 (39.4–44.1)	45.3 ± 16.8 (44.7–45.9)	48.6 ± 16.0 (48.2–49.0)	47.2 ± 16.2 (46.5–47.9)	41.4 ± 17.0 (40.9–41.9)	<0.001
**BMI:** mean ± SD a		25.8 ± 4.8 (25.2–26.5)	25.2 ± 4.3 (25.1–25.4)	25.7 ± 4.4 (25.6–25.8)	26.4 ± 4.7 (26.3–26.6)	25.0 ± 4.6 (24.9–25.1)	<0.001
**Sex**^b^							
	Male	56.5 (49.8–63.1)	48.8 (47.0–50.5)	51.8 (50.6–53.0)	61.0 (59.0–63.1)	42.0 (40.4–43.7)	
	Female	43.5 (36.9–50.2)	51.2 (49.5–53.0)	48.2 (47.0–49.4)	39.0 (36.9–41.0)	58.0 (56.3–59.6)	
**Socioeconomic status (SES)**^b^							<0.001
	High non-manual	7.6 (4.4–10.9)	12.1 (11.0–13.2)	9.3 (8.6–9.9)	6.6 (5.6–7.6)	7.8 (7.1–8.6)	
	Medium non-manual	11.4 (7.6–15.2)	16.7 (15.5–17.9)	14.5 (13.6–15.3)	12.2 (11.0–13.5)	12.4 (11.4–13.4)	
	Low non-manual	7.1 (3.1–11.1)	8.3 (7.4–9.3)	8.2 (7.5–8.9)	6.2 (5.2–7.2)	8.6 (7.7–9.4)	
	Skilled manual	6.5 (3.0–9.9)	9.4 (8.4–10.4)	10.7 (10.0–11.4)	12.6 (11.2–14.0)	10.4 (9.4–11.4)	
	Unskilled manual	16.6 (10.9–22.3)	10.4 (9.3–11.5)	12.2 (11.4–13.0)	13.8 (12.3–15.2)	14.3 (13.2–15.4)	
	Self-employed/farmer	6.2 (2.9–9.5)	6.6 (5.7–7.4)	6.0 (5.4–6.6)	6.7 (5.6–7.7)	5.4 (4.7–6.2)	
	Early retired	4.0 (1.3–6.7)	2.5 (2.0–3.0)	3.9 (3.4–4.4)	5.6 (4.7–6.6)	3.4 (2.9–4.0)	
	Unemployed	5.8 (2.5–9.0)	2.8 (2.1–4.0)	3.3 (2.8–3.8)	4.6 (3.7–5.6)	5.4 (4.5–6.2)	
	Student	14.0 (8.6–19.3)	9.4 (8.3–10.5)	6.6 (5.9–7.3)	5.8 (4.7–6.9)	11.2 (10.2–12.3)	
	Old age pensioner	12.3 (8.4–16.1)	16.0 (14.9–17.1)	20.3 (19.4–21.2)	18.0 (16.6–19.3)	12.4 (11.5–13.3)	
	Unclassified	8.1 (4.1–12.1)	5.1 (4.2–6.0)	4.1 (3.6–4.7)	6.2 (5.0–7.3)	7.1 (6.2–8.1)	
	Long-term sick leave	0.5 (0.0–1.2)	0.7 (0.4–1.0)	1.0 (0.7–1.2)	1.7 (1.2–2.3)	1.5 (1.1–1.9)	
**Country of birth**^b^		29.5 (22.4–36.6)	17.5 (16.0–19.0)	15.3 (14.2–16.3)	16.0 (14.2–17.7)	21.5 (20.1–23.0)	<0.001
**Chronic disease**^b^		23.1 (17.5–28.7)	23.0 (21.6–24.4)	29.8 (28.7–30.9)	36.1 (34.2–38.1)	26.3 (24.9–27.7)	<0.001
**Low LTPA**^b^		19.1 (13.5–24.7)	10.3 (9.2–11.4)	13.0 (12.1–13.8)	17.8 (16.2–19.5)	15.7 (14.5–16.9)	<0.001
**Daily smoking**^b^		10.5 (6.2–14.7)	10.8 (9.6–11.9)	14.0 (13.1–14.9)	18.3 (16.6–19.9)	15.6 (14.4–16.9)	<0.001
**Alcohol drinking past year**^b^							<0.001
Never		26.1 (19.5–32.8)	10.3 (9.2–11.4)	9.8 (9.0–10.6)	12.5 (11.1–13.8)	13.2 (12.0–14.3)	
Once a month or more seldom		19.8 (14.7–25.0)	19.8 (18.4–21.2)	22.5 (21.5–23.6)	24.1 (22.3–25.9)	24.8 (23.5–26.2)	
2–4 times a month		31.9 (25.6–38.2)	36.5 (34.8–38.1)	35.7 (34.5–36.9)	34.9 (32.9–36.8)	36.9 (35.3–38.5)	
2–3 times a week		18.0 (12.4–23.7)	26.2 (24.7–27.6)	23.9 (22.8–24.9)	20.3 (18.6–21.9)	19.5 (18.2–20.8)	
At least 4 times a week		4.1 (2.0–6.2)	7.3 (6.5–8.1)	8.1 (7.5–8.8)	8.3 (7.2–9.4)	5.6 (4.9–6.3)	
**Low generalized trust in other people**^b^							<0.001
		27.5 (20.7–34.2)	23.9 (22.4–25.4)	36.6 (35.4–37.8)	51.1 (49.0–53.1)	39.0 (37.4–40.6)	

The values in parentheses are 95% confidence intervals for mean or percent based on bootstrap method with 1000 number of replicates.

a p-value: Independent samples ANOVA-test, 2-tailed.

b p-value: Pearson Chi Square test, 2-sided.

**Table 2:** **Crude model**. Hazard rate ratios (HRRs) with 95% confidence intervals (95% CIs) of all-cause mortality. The 2008–2016 Scania public health survey with 8.3 years follow-up. Men and women combined. Total population **n = 24699**. **Weighted prevalence.**

			**Crude**		
**Cause of death**		**Ref.**	**HR**	**(95% CI)**	**Number of** **deaths**
**All causes**					1241
**Trust in regional politicians**		Very high trust			
Rather high trust			0.6	(0.4–1.1)	
Not particularly high			0.9	(0.5–1.5)	
No trust			1.0	(6–1.8)	
Don’t know			0.7	(0.4–1.2)	
**Sex**		Male	0.6***	(0.5–0.7)	
**Age**			1.1***	(1.1–1.1)	
**BMI**			1.0***	(1.0–1.1)	
**Socioeconomic status**		High non-manual			
	Medium non-manual		0.9	(0.5–1.6)	
	Low non-manual		1.2	(0.6–2.3)	
	Skilled manual		1.1	(0.6–2.1)	
	Unskilled manual		1.0	(0.5–1.7)	
	Self-employed/farmer		1.1	(0.6–2.3)	
	Early retired		11.0***	(6.3–19.3)	
	Unemployed		1.5	(0.7–3.0)	
	Student		0.2	(0.0–1.2)	
	Old age pensioner		17.1***	(10.4–28.2)	
	Unclassified		0.6	(0.2–1.8)	
	Long-term sick leave		8.9***	(4.5–17.9)	
**Country of birth**		Swedish	0.9	(0.7–1.1)	
**Chronic disease**		No	3.2***	(2.8–3.7)	
**Leisure-time physical activity**		Active	2.7***	(2.4–3.1)	
**Daily smoking**		No	1.6***	(1.3–1.9)	
**Alcohol drinking past year**		Never			
	Once a month or more seldom		0.7**	(0.6–0.9)	
	2–4 times a month		0.3***	(0.3–0.4)	
	2–3 times a week		0.5***	(0.4–0.7)	
	At least 4 times a week		1.2	(1.0–1.6)	
	**Generalized trust in other people**	High			
	Low		1.1	(1.0–1.3)	

Significance levels: * p < 0.05, ** p < 0.01, *** p < 0.001. Weighted Hazard Ratios. Bootstrap method (1000 replicates) for variation estimation.

**Table 3:** Hazard rate ratios (HRRs) and 95% confidence intervals (95% CIs) from Cox regression models for all-cause and cause-specific mortality according to trust in regional politicians. The 2008–2016 Scania public health survey with 8.3 years follow-up. Men and women combined. Total population n = 24699 (Men = 11245 and women = 13454). **Weighted prevalence.**

	**Model 0**		**Model 1**		**Model 2**		**Model 3**		**Model 4**		
**Cause of death**	**HR**	**(95 %CI)**	**HR**	**(95 %CI)**	**HR**	**(95 %CI)**	**HR**	**(95 %CI)**	**HR**	**(95% CI)**	**Number of** **deaths**
**All causes**											1241
Very high trust = Ref	1.0		1.0		1.0		1.0		1.0		
Rather high trust	0.6	(0.4–1.1)	0.5*	(0.3–0.9)	0.5**	(0.3–0.8)	0.6*	(0.3–1.0)	0.6*	(0.3–1.0)	
Not particularly high	0.9	(0.5–1.5)	0.6	(0.4–1.0)	0.5*	(0.3–0.9)	0.6*	(0.3–1.0)	0.6*	(0.3–1.0)	
No trust	1.0	(0.6–1.8)	0.8	(0.5–1.4)	0.6	(0.4–1.1)	0.7	(0.4–1.2)	0.7	(0.4–1.1)	
Don’t know	0.7	(0.4–1.2)	0.8	(0.5–1.3)	0.7	(0.4–1.2)	0.7	(0.4–1.2)	0.7	(0.4–1.2)	
**Cardiovascular disease**											366
Very high trust = Ref	1.0		1.0		1.0		1.0		1.0		
Rather high trust	0.4	(0.1–1.3)	0.4	(0.1–1.1)	0.3*	(0.1–1.0)	0.4	(0.1–1.2)	0.4	(0.1–1.2)	
Not particularly high	0.6	(0.2–1.8)	0.5	(0.2–1.4)	0.4	(0.1–1.1)	0.5	(0.2–1.4)	0.5	(0.2–1.3)	
No trust	0.8	(0.3–2.2)	0.7	(0.2–1.9)	0.5	(0.2–1.5)	0.5	(0.2–1.6)	0.5	(0.2–1.6)	
Don’t know	0.6	(0.2–1.6)	0.8	(0.3–2.2)	0.6	(0.2–1.8)	0.7	(0.2–1.9)	0.7	(0.2–1.9)	
**Cancer**											492
Very high trust = Ref	1.0		1.0		1.0		1.0		1.0		
Rather high trust	1.0	(0.2–5.0)	0.8	(0.2–3.9)	0.7	(0.1–3.8)	0.8	(0.2–4.0)	0.8	(0.2–4.0)	
Not particularly high	1.2	(0.2–6.1)	0.8	(0.2–4.2)	0.8	(0.2–3.9)	0.8	(0.2–4.0)	0.8	(0.2–3.9)	
No trust	1.4	(0.3–7.1)	1.1	(0.2–5.5)	1.0	(0.2–4.8)	0.9	(0.2–4.8)	0.9	(0.2–4.7)	
Don’t know	1.0	(0.2–5.1)	1.1	(0.2–5.6)	1.0	(0.2–5.2)	1.0	(0.2–5.2)	1.0	(0.2–5.2)	
**Others**											383
Very high trust = Ref	1.0		1.0		1.0		1.0		1.0		
Rather high trust	0.6	(0.1–3.2)	0.5	(0.1–2.6)	0.5	(0.1–2.4)	0.6	(0.1–2.9)	0.6	(0.1–2.9)	
Not particularly high	0.8	(0.2–4.1)	0.6	(0.1–2.9)	0.5	(0.1–2.5)	0.6	(0.1–2.9)	0.6	(0.1–2.9)	
No trust	0.9	(0.2–4.9)	0.7	(0.1–3.9)	0.6	(0.1–3.0)	0.6	(0.1–3.2)	0.6	(0.1–3.2)	
Don’t know	0.5	(0.1–2.6)	0.6	(0.1–3.1)	0.5	(0.1–2.5)	0.5	(0.1–2.7)	0.5	(0.1–2.6)	

Model 0 Unadjusted.

Model 1 Adjusted for sex and age.

Model 2 Additionally adjusted for socioeconomic status (SES), country of birth and chronic disease.

Model 3 Additionally adjusted for BMI, leisure-time physical activity (LTPA), smoking and alcohol drinking past year.

Model 4 Additionally adjusted for generalized trust in other people.

Significance levels: * p < 0.05, ** p < 0.01, *** p < 0.001.

Weighted Hazard Ratios. Bootstrap method (1000 replicates) for variation estimation.

*Age* was included and analyzed as a continuous variable.

*Country of birth* was defined as born in Sweden or born in other country.

*Socioeconomic status (SES)* was classified as non-manual employees in high, medium and low work positions, skilled and unskilled manual workers, and self-employed/farmers. SES groups outside the active workforce include the unemployed (registered as job seekers), students, early retired (before age 65), long-term sick leave, pensioners aged 65-, and unclassified.

*Chronic disease* was measured with the question “Do you have any long-term disease, ailment or injury, any disability or other weakness?”, with the alternatives “Yes” and “No”.

*Body mass index (BMI)* was assessed by self-report of weight (kg) and height (m) in the public health survey in 2008, and analyzed as a continuous variable.

*Leisure-time physical activity* (LTPA) was assessed with an item including the four alternative**s** regular, moderate regular and moderate exercise, and low LTPA (<2 h walking, cycling or equivalent activity/week). The three first alternatives were collapsed and defined as high LTPA and the sedentary alternative was defined as low LTPA (see Lindström et al., 2021).

*Daily smoking* was assessed with the item “Do you smoke?” with the alternatives daily, non-daily and non-smoker. The two latter alternatives were collapsed.

*Alcohol consumption* was measured with the question “How often have you consumed alcohol during the past twelve months?”, which included the alternative answers “Four times per week or more”, “2–3 times per week”, “2–4 times per month”, “Once per month or more seldom”, and “Never”.

*Generalized trust in other people* was measured with the question “Most people can be trusted”. The optional answers were “Do not agree at all”, “Do not agree”, “Agree” and “Agree completely”. The item was dichotomized with the two first defined as “low trust” and the two latter as “high trust”.

### 2.4 Statistics

Prevalence (%) of all items were analyzed stratified by the five alternative answers to the trust in the regional politicians item. Differences for each variable according to the five trust in the regional politicians alternatives were analyzed using ANOVA tests for continuous variables and chi-square tests for categorical variables (p-values) (Table 1). Hazard rate ratios (HRRs) and 95% confidence intervals (95% CIs) were calculated for univariate associations between independent 2008 questionnaire variables and all-cause mortality (Table 2). HRRs and 95% CIs of all-cause, CVD, cancer and other causes mortality were calculated according to trust in regional politicians with very high trust defined as reference category. Five models 0–4 were investigated: model 0 unadjusted, model 1 adjusted for age and sex, model 2 adjusted for age, sex, country of birth, SES, and chronic disease, model 3 additionally adjusted for BMI, LTPA, smoking and alcohol consumption, and model 4 additionally adjusted for generalized trust in others (Table 3). Follow-up days were measured from the survey baseline in the autumn of 2008 to death or last date of follow-up (31 December 2016). Sampling variability may be investigated without assumptions with regard to the distribution of the study population using bootstrap analysis (SAS/STAT Software Survey Analysis, 2021). Bootstrap methods with 1000 numbers of replicates to obtain confidence intervals and p-values were used in order to ensure accurate variance estimation on weighted data. Tests of proportionality for trust in regional politicians and mortality were performed in order to ascertain that the application of the survival (Cox) regression models was statistically correct. An interaction term with time and trust in regional politicians was introduced to test the assumption of proportional hazards. Schoenfeld residuals were estimated for trust in regional politicians and mortality as an illustration of proportionality and consistency of the association between political trust and mortality. The Schoenfeld residuals compare the respondent categories with very high and rather high trust in regional politicians to the two collapsed respondent categories with not particularly high and no trust in the regional politicians (Fig. 1). The SAS software version 9.4 was used for the calculations.

## 3 Results

Table 1 shows that 1.4% had very high trust, 20.3% rather high trust, 39.3% not particularly high trust, 14.2% no trust in regional politicians, while 24.8% didn’t know. The respondent category who reported no trust in regional politicians had significantly higher BMI than respondent categories with any other answer to the question regarding trust in regional politicians. Men were particularly overrepresented in the category reporting no trust in regional politicians, while women were overrepresented in the category reporting that they didn’t know. Non-manual employees in higher and medium positions reported rather high trust in regional politicians to a significantly higher extent than any other political trust category. Pensioners aged 65 years and above reported not particularly high trust in regional politicians to a significantly higher extent than the other alternatives. Respondents born in other countries than Sweden were significantly overrepresented in the category reporting very high trust and the category reporting “don’t know”. The respondent category with no trust in regional politicians had a significantly higher prevalence of self-reported chronic disease. The respondent category with rather high trust in regional politicians had a significantly lower prevalence of low LTPA than the other categories. Respondents with no trust in regional politicians were smokers to a significantly higher extent. The respondent category with very high trust in regional politicians had a significantly higher prevalence of having never consumed alcohol. Respondents with no trust in regional politicians reported low generalized trust to a significantly higher extent than all other categories.

Table 2 shows positive univariate associations between male sex, higher age, higher BMI, early retired, pensioner, long-term sick leave, chronic disease, low LTPA and smoking, and all-cause mortality, compared to the respective reference groups. Lower moderate frequency levels of alcohol consumption were associated with lower all-cause mortality.

Table 3 shows that the categories with rather high trust and not particularly high trust in regional politicians had significantly lower HRRs of all-cause mortality than the very high trust category. Respondents with rather high trust in regional politicians had a HRR 0.5 (0.3–0.9) of all-cause mortality compared to the very high reference category in the sex-and age-adjusted model 1, and this significant association persisted with a HRR 0.6 (0.3–1.0) in the final multiple adjusted model 4. For the not particularly high trust in regional politicians respondent category a statistically not significant HRR 0.6 (0.4–1.0) in the sex-and age-adjusted model 1 was followed by a HRR 0.5 (0.3–0.9) in model 2. This pattern remained significant throughout the following multiple analyses with a HRR 0.6 (0.3–1.0) in the final multiple adjusted model 4. CVD, cancer and other causes mortality all seem to contribute to this pattern for all-cause mortality, although each of them individually were not statistically significant. No statistically significant results were observed for the no trust in regional politicians and don’t know categories.

Fig. 1 shows consistent and stable Schoenfeld residuals over time for trust in regional politicians and all-cause mortality when the two collapsed respondent categories with very high and rather high trust in regional politicians were compared with the two collapsed categories with not particularly high trust and no trust in regional politicians. The interaction term between trust in the regional politicians and all-cause mortality over the 8.3 year period was p = 0.436, which indicates proportionality.

## 4 Discussion

All-cause mortality was lower for the respondent categories with rather high trust and not particularly high trust in regional politicians compared to the very high trust category. The broad cause-specific categories CVD, cancer and other causes mortality did not display statistically significant results, but all seem to contribute to the significant patterns in all-cause mortality with effect measures (HHRs) below 1.00. Depending on political and administrative settings, rather high and not particularly high trust may be associated with lower mortality than very high trust in regional politicians responsible for the healthcare system. The second hypothesis suggesting lower mortality for somewhat lower levels of trust compared to very high trust was confirmed.

Trust has a special connotation. Generalized trust means the expectation that other persons will fulfill the expectations directed towards them. Institutional (vertical) trust means that institutions and their leaderships will deliver according to expectations (Baier, 1986). Political trust may be regarded as a combination of trust in the political system in general and the current incumbent politicians (Hetherington, 1998), but the item in this study specifically point**s** to incumbents. The fact that only 1.4% express very high trust and 20.3% rather high trust in regional politicians, while 39.3% express not particularly high trust and 14.2% no trust, points to the growing systemic problems in the Swedish healthcare system. These problems are systemic because they are related to political leadership, not primarily to problems with individual caregivers delivering healthcare to patients. The low prevalence of very high political trust in the Scania population runs counter to the notion that heuristic behavior and the use of cognitive shortcuts based on lazy and unconditioned trust would be widespread (Myskja et al., 2020).

The problems with queueing for treatments and planned operations, particularly (but not only) for several important cancer diagnoses, existed at the time of the public health survey in 2008 but have grown since then during the following decade. As a result, patients in Scania have been medically treated in other countries (Dagens Medicin, 2019). Private alternatives have emerged for groups who can afford insurances or can afford to pay outside the publicly tax-financed insurance system (Trysell, 2020). Parts of the primary healthcare system have been privatized (Lindström et al., 2018). It may be that in a system of scarce healthcare resources and queueing for essential treatments rather high trust and not particularly high trust in regional politicians responsible for healthcare may be more rational than very high levels of trust. Critical assessment and questioning may promote better solutions for patients. Institutional trust promotes political debate, discussion, problem solving and cooperation, but democracy is also based on light to moderate distrust and questioning of decision makers (Uslaner, 2002). Our previous study of trust in the healthcare system and mortality supports this notion (Lindström and Pirouzifard, 2022). The present study shows diluted but similar significant associations between trust in regional healthcare politicians and mortality. These results give a modified and more complex picture of the association between political trust and health compared to the general literature, which mostly indicates significant associations between high political trust and better health and lower mortality (Bollyky et al., 2022, Charron et al., 2022).

The 1.4% prevalence of very high trust and the comparatively low prevalence 20.3% of rather high trust in regional politicians in 2008 is notable. Trust in the national parliament (*Riksdag*) and national politicians in Sweden continually declined from the 1950 s until the 1990s, and reached a bottom low in the late 1990s (Holmberg, 1999, Rothstein, 2001). Since the late 1990s, an increase in trust in the national parliament and national politicians partly back to “normalcy” occurred (Oscarsson and Holmberg, 2016). Contrary to what might be a priori expected, the public’s political trust has for decades been systematically higher in the national parliament and politicians than in municipal politicians. A priori hypotheses suggesting that trust would be higher in local politicians closer to the respondent**s** have been contradicted (Erlingsson, 2022). The results of this study indicate that similar patterns of lower political trust below the national level may also exist for the regional level. In fact, in the 2008 public health survey 4.7% of respondents reported very high trust and 36.1% rather high trust in the national parliament (*Riksdag*), while only 1.4% reported very high trust and 20.3% rather high trust in regional politicians, and 1.9% reported very high trust and 24.0% rather high trust in municipal politicians. These results confirm previous political science research.

### 4.1 Strengths and limitations

This study is a large and population-based prospective cohort study. The response rate 54.1% is comparable to results in Sweden and other countries with declining response rates. The study population has acceptable representativeness regarding sex, age, country of birth and education, although some underrepresentation of men, young adults, born abroad and respondents with low education is observed. Consequently, selection bias is less likely in the 2008 public health survey (Lindström and Rosvall, 2018).

Items regarding political trust have mostly been analyzed in the general social capital literature (Putnam, 1993, Holmberg, 1999) and much more scarcely in the public health literature (Mohseni and Lindström, 2008, Lindström and Mohseni, 2009). Only few health studies have included items on political trust, which makes our study unique in its approach. SES has been defined according to occupation, education and income. These dimensions are correlated but not identical. The 2008 public health survey entailed no items regarding income, and included an item regarding education that does not alter the associations, but has a substantially higher number of internally missing. Compared to golden standard items the LTPA item is regarded as valid (Wareham et al., 2003). Smoking items are valid (Wells et al., 1998). With the three broad diagnosis-specific mortality groups misclassification is less likely.

Adjustments for relevant cofounders/covariates were made in the multiple survival analyses. All independent variables from the questionnaire were measured in 2008.

## 5 Conclusion

Hazard rate ratios of all-cause mortality were consistently lower for the rather high trust and not particularly high trust in regional politicians respondent categories compared to the very high trust reference. CVD, cancer and other cause mortality did not display statistically significant results, but all seem to have contributed to the significant patterns in all-cause mortality. Under certain political and administrative circumstances, rather high and not particularly high trust in regional politicians responsible for healthcare may be associated with lower mortality compared to very high trust.

## Funding

The present study was approved by the Ethical Committee (*Etikprövningsnämnden*) in Lund (No. 2010/343).

## Declaration of Competing Interest

The authors declare that they have no known competing financial interests or personal relationships that could have appeared to influence the work reported in this paper.

## Data Availability

The authors do not have permission to share data.
